# Pulmonary Nocardiosis Caused by Nocardia sputorum Identified via 16S rRNA Gene Sequencing: A Case Report

**DOI:** 10.7759/cureus.66137

**Published:** 2024-08-04

**Authors:** Satoshi Irifune, Shotaro Ide, Satoru Koga, Keisuke Mine, Nanae Sugasaki, Kosuke Kosai, Minoru Fukuda, Katsunori Yanagihara, Hiroshi Mukae

**Affiliations:** 1 Department of Respiratory Medicine, Nagasaki University Graduate School of Biomedical Sciences, Nagasaki, JPN; 2 Department of Respiratory Medicine, Nagasaki Prefecture Shimabara Hospital, Nagasaki, JPN; 3 Infectious Diseases Experts Training Center, Nagasaki University Hospital, Nagasaki, JPN; 4 Department of Respiratory Medicine, Nagasaki University Hospital, Nagasaki, JPN; 5 Department of Laboratory Medicine, Nagasaki University Hospital, Nagasaki, JPN; 6 Department of Respiratory Medicine, Japanese Red Cross Nagasaki Genbaku Isahaya Hospital, Nagasaki, JPN

**Keywords:** anaerobe, anaerobic bacteria, 16s rrna gene, nocardia sputorum, nocardiosis, pulmonary infection

## Abstract

*Nocardia sputorum*, a novel *Nocardia* species discovered in Japan in 2023, has not been reported to infect humans. Here, we report a case of pulmonary nocardiosis in a 70-year-old immunocompetent woman infected with *N. sputorum*. The patient presented to the hospital with a chief complaint of weight loss. She worked at a fruit sorting facility where she was exposed to dust. Chest computed tomography revealed a single cavity and diffuse nodular opacities in both lungs. *Nocardia* species was isolated from tracheal sputum and bronchial lavage fluid and identified as *N. sputorum* via 16S rRNA gene sequencing. The patient was treated with oral sulfamethoxazole and trimethoprim but developed oral mucositis on the 12th day of treatment. Consequently, minocycline was prescribed, and the patient's condition improved after a six-month course of treatment. To our knowledge, this is the first reported case of pulmonary nocardiosis caused by *N. sputorum* in humans. Accurate species identification and antimicrobial susceptibility tests will be necessary to prescribe appropriate treatment for *Nocardia *infections.

## Introduction

*Nocardia* are gram-positive to gram-variable, aerobic, and acid-fast filamentous branching bacteria belonging to the family *Nocardiaceae*. They are ubiquitous in various environments, such as soil, vegetables, other plants, and water. *Nocardia* causes acute or chronic infections in humans affecting the lungs, central nervous system, and skin [1]. These infections are typically opportunistic; however, it is estimated that one-third of the affected individuals are immunocompetent [1,2]. Novel species of the genus *Nocardia* have been discovered via molecular biology methods; more than 100 species have been identified in total, approximately half of which are pathogenic to humans [3,4]. *Nocardia sputorum* is a novel species isolated from clinical specimens in Japan in 2023; however, its pathogenicity in humans has not been reported [5]. To the best of our knowledge, we report the first case of pulmonary nocardiosis caused by *N. sputorum* in an immunocompetent patient.

## Case presentation

**Figure 1:**
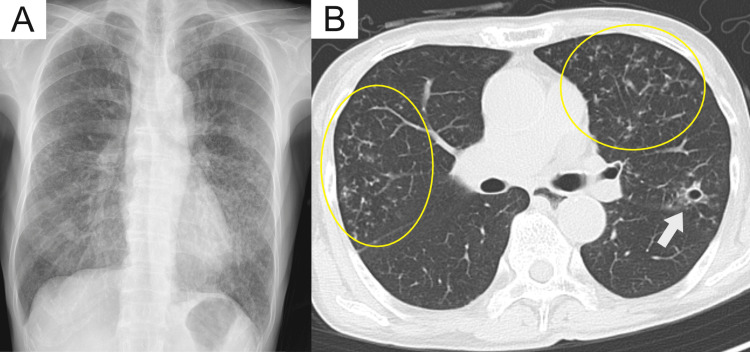
Chest radiograph and computed tomography A: Chest radiograph shows diffuse nodular opacities; B: Chest computed tomography shows a cavitary lesion (white arrows) in the left upper lobe and bilateral centrilobular bronchiolitis (yellow circles).

A 70-year-old woman presented to our hospital with a weight loss of 10 kg over the past six months without any other symptoms, including fever, cough, sputum production, or shortness of breath. The patient was employed at a fruit sorting facility, where she was frequently exposed to dust without wearing a protective mask. Physical examination revealed clear breath sounds, absence of skin rash, and absence of neurological signs. Blood tests showed a white blood cell count of 8,100 cells/µL, a neutrophil count of 6,100 cells/µL, a lymphocyte count of 1,400 cells/µL, a C-reactive protein level of 3.23 mg/dL, negative human immunodeficiency virus antigen/antibody test results, and negative blood culture results. Chest radiography revealed diffuse nodular opacities in both lungs (Figure 1A), and chest computed tomography (CT) revealed multiple diffuse centrilobular nodules in both lungs along with a small cavity (Figure 1B).

**Figure 2:**
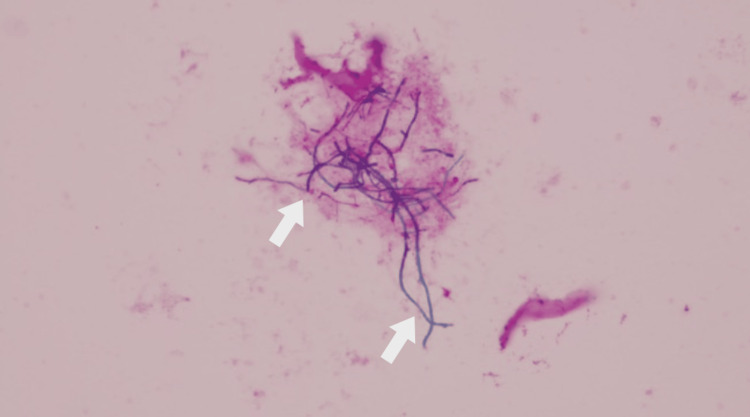
Gram staining for isolates from tracheal sputum and bronchoalveolar fluid show a gram-positive and filament-like bacterium

The presence of cavities led to the differential diagnosis of pulmonary tuberculosis and nontuberculous mycobacteriosis; however, sputum cultures did not contain mycobacteria, bacteria, or fungi. Bronchoscopy was performed, followed by sputum collection from the trachea and bronchial lavage. Gram and Kinyoun staining for these samples revealed the presence of a gram-positive, weak acid-fast, and filament-like bacterium in smears and cultures (Figure 2), which was identified as a *Nocardia* sp. via matrix-assisted laser desorption/ionization time-of-flight mass spectrometry (MALDI-TOF MS).

Subsequently, further testing was performed using 16S rRNA gene sequencing at the Nagasaki University Hospital, Nagasaki, Japan. The 16S rRNA gene was amplified using the primers 8UA (5′-AGAGAGTTTGATCMTGGCTCAG-3′) and 1485B (5́’-ACGGGCGGTGTGTRC-3′) [6]. The nucleotide sequence was determined and searched against the BLAST database (https://blast.ncbi.nlm.nih.gov/Blast.cgi), and it showed 100% nucleotide identity (1,243/1,243) with *N. sputorum* [7]. Brain CT and contrast-enhanced magnetic resonance imaging showed no evidence of brain abscess; thus, the patient was diagnosed with pulmonary nocardiosis. The patient was treated with oral sulfamethoxazole and trimethoprim (ST) based on antimicrobial susceptibility testing results (Table 1) [8].

**Table 1:** Antimicrobial susceptibility testing for Nocardia sputorum MIC: minimum inhibitory concentration; S: sensitive; I: intermediate resistance; R: resistance

Antimicrobial agent	MIC (µg/mL) for category [8]			Present case		Hamada et al. [5] (2 strains)
	S	I	R			
Amikacin	≤8	-	≥16	≤4	S	S
Amoxicillin-clavulanic acid	≤8/4	16/8	≥32/16	≤4	S	S
Ceftriaxone	≤8	16–32	≥64	8	S	-
Ciprofloxacin	≤1	2	≥4	-	-	R
Clarithromycin	≤2	4	≥8	≤1	S	R
Imipenem	≤4	8	≥16	>16	R	R
Linezolid	≤8	-	-	≤2	S	S
Minocycline	≤1	2–4	≥8	1	S	-
Moxifloxacin	≤1	2	≥4	≤0.5	S	S
Trimethoprim-Sulfamethoxazole	≤2/38	-	≥4/76	≤0.5	S	S
Tobramycin	≤4	8	≥16	≤1	S	S
Cefotaxime	≤8	16–32	≥64	≤1	S	-

**Figure 3:**
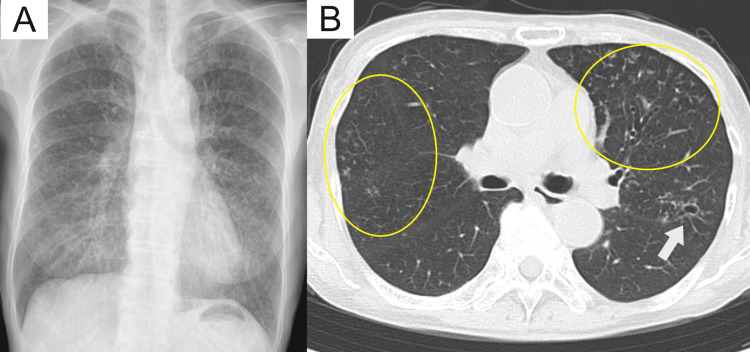
Chest radiograph and computed tomography after six months of treatment A: Chest radiograph shows improvement in diffuse nodular opacities; B: Chest computed tomography shows improvement in cavitary lesion (white arrows) and centrilobular nodules (yellow circles).

However, on the 12th day of treatment, the patient exhibited grade 3 oral mucositis based on the Common Terminology Criteria for Adverse Events version 5.0. Consequently, ST treatment was discontinued, and minocycline (MINO) was prescribed. Following a six-month course of treatment, weight loss improved in the patient, and chest CT revealed improvement in centrilobular nodules in both lungs (Figure 3A, 3B). The patient did not exhibit any recurrence at the three-month follow-up after treatment termination.

## Discussion

To the best of our knowledge, this case report describes the first documented infection of *N. sputorum* in humans. *Nocardia* are gram-positive aerobic bacilli found in soil, plants, and water. Molecular biological tools have led to the discovery of novel species within the genus *Nocardia*; 130 species have been validly published at the time of this study [9,10]. The prevalence of *Nocardia* species varies with geographical location. *Nocardia nova* complex was the most commonly isolated clinical strain between 1995 and 2004 in the United States, comprising 28% of the total, followed by *Nocardia brasiliensis* and *Nocardia farcinica*, which accounted for 14% of the isolates [11]. *Nocardia cyriacigeorgica* and *N. nova* complex were the dominant clinical isolates between 2020 and 2021 in Western Australia, representing 27% and 20% of the total, respectively; however, there were regional differences [12]. Takamatsu et al. (2022) reported that *N. farcinica* was the most commonly identified clinical strain in Japan between 2010 and 2017, accounting for 24.9% of the isolates, followed by *N. nova *complex (19.2%) and *Nocardia abscessus* complex (18.6%) [13]. From 2006 to 2021, 48 novel *Nocardia* species were identified, 17 of which were obtained from human specimens [4]. Furthermore, pathogenic *Nocardia* species that infect humans have been reported in recent years [14-17].

*N. sputorum* is a recently discovered species isolated from a clinical specimen, and it was first reported by Hamada et al. (2023) in Japan in 2023 [5]. *N. sputorum* exhibited high similarity to *Nocardia beijingensis* (99.6%), *Nocardia sputi *(99.6%), *Nocardia niwae *(99.3%), and *Nocardia araoensis* (99.3%) based on 16S rRNA gene sequencing. However, the phenotypic characteristics, DNA-DNA hybridization analysis, and genome sequencing distinguished it as a novel species within the genus *Nocardia*, and this has been validly published under the International Code of Nomenclature of Prokaryotes [9,10]. Two strains were previously isolated from the sputum and a clinical sample of unknown origin; however, their pathogenicity in humans has not yet been reported. According to us, this case report is the first to report the pathogenicity of *N. sputorum* in humans based on a pulmonary infection. MALDI-TOF MS and 16S rRNA gene sequencing have been used to identify *Nocardia*; however, the use of MALDI-TOF MS can lead to misidentification [18]. *N. sputorum* may be misidentified as other *Nocardia* species such as *N. beijingensis*,* N. sputi*, *N. niwae*, and *N. araoensis*. In contrast, 16S rRNA gene sequencing is useful for accurate identification of *Nocardia* species. The *Nocardia* species isolated from our patient was unidentifiable via MALDI-TOF MS and required identification using 16S rRNA gene sequencing.

Although pulmonary nocardiosis is relatively more common in immunocompromised patients, it also affects healthy individuals without immunodeficiency [1,2]. The present case reported pulmonary infection in an immunocompetent host, and radiological findings revealed centrilobular nodular opacities and cavitary lesions. The possible source of infection was considered to be contact with contaminated vegetables in the selection area and inhalation of soil dust. However, the isolation of *N. sputorum* from soil has not been reported.

No standard treatment regimen has been established for nocardiosis, and antibiotics and treatment durations are customized according to the infection site, underlying disease, severity, and antimicrobial susceptibility testing [1,4]. Several *Nocardia* species are susceptible to ST, which is used as a first-line drug [13,19]. However, certain *Nocardia* species are resistant to ST; therefore, accurate species identification and antimicrobial susceptibility testing are necessary. The strain isolated from this patient and the two strains reported by Hamada et al. (2023) were found to be susceptible to ST, amoxicillin-clavulanic acid, MINO, and moxifloxacin while displaying resistance to imipenem. Conversely, our strains were susceptible to clarithromycin, with the latter two strains exhibiting resistance. *N. beijingensis*, which *N. sputorum* was likely misidentified, is included in *N. abscessus* complex, and the susceptibility rates to ST, amoxicillin-clavulanic acid, MINO, moxifloxacin, clarithromycin, and imipenem were reported as 98.7-100%, 26.3%, 67-98.7%, 22%, 17-51.3%, and 72-88.2%, respectively [19,20]. These differences highlight the importance of species identification. The patient was initially administered ST, which was discontinued due to grade 3 mucositis. Since no bacteria other than *N. sputorum* were detected in this patient and there was no evidence of central nervous system infection, treatment was switched to MINO based on antimicrobial susceptibility testing and antimicrobial spectrum, and a favorable response was observed.

To the best of our knowledge, this is the first report of pulmonary nocardiosis caused by *N. sputorum*, a novel species reported in 2023, which may have been previously misidentified as *N. beijingensis* or *N. sputi*. Additional cases need to be evaluated to determine the clinical features of *N. sputorum* infection and appropriate antimicrobial therapy.

## Conclusions

To the best of our knowledge, this report presents the first documented case of human infection caused by *Nocardia sputorum*, a novel species of *Nocardia* discovered in Japan in 2023. Differences in the clinical course and characteristics of nocardiosis depending on the species are unknown. In contrast, *Nocardia* species exhibit a variety of antimicrobial susceptibilities. This case emphasizes the significance of precise species identification and antimicrobial susceptibility testing for effective treatment of *Nocardia* infections. Further cases need to be assessed to determine the clinical characteristics of *N. sputorum* infection and the appropriate antimicrobial therapy.
